# Time dependent modulation of tumor radiosensitivity by a pan HDAC inhibitor: abexinostat

**DOI:** 10.18632/oncotarget.14813

**Published:** 2017-01-25

**Authors:** Sofia Rivera, Céline Leteur, Frédérique Mégnin, Frédéric Law, Isabelle Martins, Ioana Kloos, Stéphane Depil, Nazanine Modjtahedi, Jean Luc Perfettini, Christophe Hennequin, Eric Deutsch

**Affiliations:** ^1^ Department of Radiotherapy, Gustave-Roussy Cancer Campus, Villejuif, France; ^2^ INSERM 1030 Molecular Radiotherapy, Villejuif, France; ^3^ Faculté de Médecine, Université Paris-Sud, Université Paris-Saclay, Le Kremlin-Bicêtre, France; ^4^ INSERM U1196/UMR9187 CMIB, Institut Curie-Recherche, Université Paris Saclay, Le Kremlin-Bicêtre, France; ^5^ IRIS: Institut de Recherches Internationales Servier, Suresnes, France; ^6^ Department of Radiation Oncology, Hôpital Saint Louis, APHP, Paris, France

**Keywords:** epigenetics, radiosensitivity modulation, HDAC inhibitors, non-small cell lung cancer, radiotherapy

## Abstract

Despite prominent role of radiotherapy in lung cancer management, there is an urgent need for strategies increasing therapeutic efficacy. Reversible epigenetic changes are promising targets for combination strategies using HDAC inhibitors (HDACi).

Here we evaluated on two NSCLC cell lines, the antitumor effect of abexinostat, a novel pan HDACi combined with irradiation *in vitro* in normoxia and hypoxia, by clonogenic assays, demonstrating that abexinostat enhances radiosensitivity in a time dependent way with mean SER10 between 1.6 and 2.5 for A549 and H460. We found, by immunofluorescence staining, flow cytometry assays and western blotting, in abexinostat treated cells, increasing radio-induced caspase dependent apoptosis and persistent DNA double-strand breaks associated with decreased DNA damage signalling and repair. Interestingly, we demonstrated on nude mice xenografts that abexinostat potentiates tumor growth delay in combined modality treatments associating not only abexinostat and irradiation but also when adding cisplatin.

Altogether, our data demonstrate *in vitro* and *in vivo* anti-tumor effect potentiation by abexinostat combined with irradiation in NSCLC. Moreover, our work suggests for the first time to our knowledge promising triple combination opportunities with HDACi, irradiation and cisplatin which deserves further investigations and could be of major interest in the treatment of NSCLC.

## INTRODUCTION

Epigenetics is a promising field of research with growing preclinical and clinical data providing new avenues for cancer treatment. In the absence of DNA sequence alteration, gene expression driven by epigenetic changes is crucial to tumor onset and progression [1–3]. Epigenetic changes such as acetylation, methylation, phosphorylation, ubiquitination and sumoylation lead to modifications of the structure of nucleosomes impacting chromatin condensation and transcription [1, 4, 5]. Histone deacetylases (HDACs) remove acetyl groups from histones, leading to a more compact form of chromatin, favoring gene expression patterns that promote tumor development. In contrast to genetic alterations, epigenetic changes are dynamic and can be reversed and therefore can be good therapeutic targets [6].

Small molecules inhibiting HDACs activity called HDAC inhibitors (HDACi) are considered of high anti-tumor potential. Some are either already approved in clinic as vorinostat (suberoylanilide hydroxamic acid, Zolinza; Merck), romidepsin (Istodax; Celegene) and belinostat (Beleodaq; Spectrum Pharmaceuticals) for T-cell lymphomas treatment or under clinical trials alone or in combination in various hematological and solid malignancies [7, 8]. More than 28 HDACi are under development [8]. Most of them target multiple HDACs which makes it difficult to identify the biological mechanisms responsible for HDACi anti-tumor effect. Eighteen HDACS grouped in 4 classes have been described in humans. Bantscheff *et al* have developed a chemoproteomic method to test the affinity of various HDACi. They have shown that HDACi activity is dependent on the macromolecular complexes in which various HDACs can reside [9]. They provided evidence in favor of a strong binding and histone hyperacetylation with pan HDACi. This suggests using pan HDAC inhibitors like vorinostat., panobinostat or the here studied abexinostat rather than more specific HDACi [9]. Selective class IIa specific HDACi have recently been developed and failed to induce significant apoptosis or gene expression changes [10]. New class I selective HDACi such as mocetinostat are under development and have shown promising apoptosis induction and broad antitumor activity spectrum [11–14]. Other selective HDACi and polypharmacological HDACi are currently under evaluation [8].

In solid tumors, HDACi used as monotherapy in early phase clinical trials have been rather disappointing [15]. More encouraging results have been reported from preclinical combination trials associating HDACi with other anti-tumor agents. *In vitro* and *in vivo*, data suggest that the anti-tumor effect of HDACi might be due to the induction of cell cycle arrest, differentiation, cell death through various mechanisms (apoptosis, autophagy) and induced alteration in DNA repair capacity [2, 3, 16, 17]. Irradiation exposure causes DNA damage either by direct effect or indirect effect through reactive oxygen species production creating DNA single and double strand breaks (SSBs, DSBs). DSBs, more substantial and potentially lethal DNA damage, can be repaired through non-homologous end joining (NHEJ) or homologous recombination (HR) mechanisms. HDACi have been reported to repress HR-related gene expression providing a strong rationale for radiosensitization using these compounds even though mechanisms of radiosensitization seem to be multiple and deserve further investigation [3, 18].

With a median 5 year survival rate about 18%, lung cancer remains the leading cause of cancer death [19]. Non-small cell lung carcinomas (NSCLC) represent 85% of lung cancers. In most cases patients are diagnosed with unresectable disease. The standard of care for locally advanced unresectable NSCLC patients relies on the combination of a platinum based doublet of chemotherapy and concomitant radiotherapy [20]. Despite improvement in radiotherapy techniques and systemic treatment, very little progress has been made over the last decades regarding the combination of drugs and radiotherapy in this setting even though there is a major room for improvement and public health issue. There is no survival advantage to date in randomized phase III trials combining targeted agents with radiotherapy or chemoradiotherapy in locally advanced NSCLC. Most attention has been focused on epidermal growth factor (EGFR) inhibitors with either monoclonal antibodies, such as cetuximab, or tyrosine kinase inhibitors (EGFR-TKIs), such as gefitinib or erlotinib [21]. After disappointing results in unselected patient populations, investigations are ongoing to combine EGFR-TKI with radiotherapy in EGFR mutated tumors [ ClinicalTrials.gov Identifier: NCT01391260; NCT01091376; NCT01822496]. In non-mutated tumors, radiotherapy and targeted agents combination options under clinical investigation are limited.

The present study investigates the effect of abexinostat, a pan HDACi, on 2 EGFR wild type NSCLC cell lines *in vitro* and *in vivo*. Our findings here demonstrate that abexinostat induces a radiosensitizing effect, and yields to an increased tumor response *in vivo*. The increase of tumor cell kill through increased apoptosis arises from an impairment of DNA repair protein levels. DNA repair impairment accounts for a marked time and schedule dependency of the combination observed *in vivo*. Moreover, our work suggest, for the first time to our knowledge, promising triple combination opportunities with HDACi, irradiation and cisplatin which deserves further investigations and could be of major interest in the treatment of NSCLC.

## RESULTS

### Abexinostat radiosensitizes NSCLC cells *in vitro* in normoxia and hypoxia

**Figure 1:**
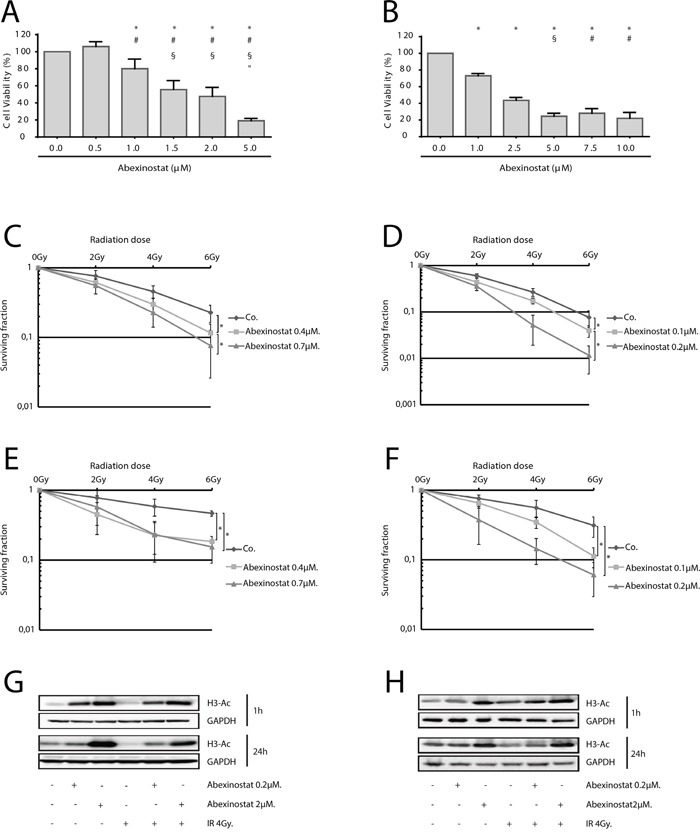
Radiosensitization of NSCLC cells by HDAC inhibition by abexinostat *in vitro* in normoxic and hypoxic conditions IC50 determined at 48h by WST-1 assay were 1.75μmol/L and 2μmol/L for A549 **A.** and H460 **B.** respectively. Results represent means ± SD (n=3; t-test; for A549 (A) ^+, #, §, ″^P < 0.05 versus control, abexinostat 0.5μM, 1μM, 1.5μM respectively; for H460 (B) ^+, #, §^P < 0.05 versus control, abexinostat 1μM, 2.5μM respectively). A549 cells **C, E, G.** and H460 cells **D, F, H.** were treated with indicated increasing concentrations of abexinostat (culture medium as control) for 24 hours then irradiated or not at indicated doses. Clonogenic radiation survival of abexinostat treated A549 (C, E) and H460 (D, F) cells in normoxic (C, D) or hypoxic (0.1% O^2^) (E, F) conditions were measured as described under materials and methods. Culture medium was used as control. Results represent means ± SD (n=3; t-test; *P < 0.05). Error bars represent standard deviations. Acetylation of histone H3 was evaluated by Western blotting 1h and 24h after irradiation in A549 (G) and H460 (H) cells. GAPDH served as loading control. Representative Western blots are shown (n=3).

A dose-dependent cell proliferation inhibition effect of abexinostat was observed in both A549 and H460 NSCLC cells (Figure 1A-1B respectively). IC50 value at 48h for A549 and H460 were 1.75μmol/L and 2μmol/L respectively, suggesting that A549 are more sensitive to abexinostat alone than H460. We tested the effect of abexinostat on clonogenic survival after irradiation in A549 and H460 cells. Exposure to abexinostat 24h before irradiation significantly decreased surviving fractions at 6Gy (SF6), for both A549 and H460, in a concentration-dependent manner, in normoxic (O^2^=21%) (Figure 1C-1D) and hypoxic conditions (O^2^=0.1% 24h before treatment as described under material and methods) (Figure 1E-1F). Corresponding surviving fractions were plotted versus dose and fit to a linear quadratic model (Supplementary Figure 1C-1F). Interestingly, SF6 was not decreased when abexinostat was given immediately before irradiation (data not shown). Calculation of α/β ratios and sensitization enhancement ratios at 10% cell survival (SER10, ratio of doses to achieve 10% cell survival without abexinostat to those with abexinostat), in normoxia and hypoxia, showed increased radiosensitivity with abexinostat, even in hypoxic condition, for both A549 and H460 cell lines (Supplementary Figure 1A-1H, Supplementary Table 1). As expected, controls with culture medium alone were less radiosensitive in hypoxia than in normoxia for both A549 and H460 cell lines (Figure 1C-1F, Supplementary Table 1). Using A549 cells, exposed to abexinostat 0.7μM starting from 24 hours before irradiation, mean SER10 varied between 1.41 in normoxia and 2.33 in hypoxia. Using H460 cells, exposed to abexinostat 0.2μM in similar conditions, mean SER varied between 1.85 in normoxia and 3.16 in hypoxia. Corresponding D10 are shown in Supplementary Figure 1A-1B with lower D10 when cells are exposed to abexinostat in normoxia and hypoxia in both cell lines. An isobologram analysis was performed as described under material and methods, for both A549 and H460 cell lines in normoxie and hypoxia (Supplementary Figure 1G-1J). In normoxia, data points for combination fell into the area of additivity for A549 cells (G) and into the supra-additivity zone for H460 cells (H). However, for this latter, the statistical test was not significant (p value = 0.25). In hypoxia, the dose-survival curves are exponential for both abexinostat alone and radiation alone as expected. In this case, the isobologram lines for Mode I and Mode II are combined and all the experimental data points for combination fell into the supra-additive zone (I,J).

Using acetyl histone H3 as a marker of HDACi inhibitory activity, we assessed Histone de-acetylation inhibitory effect of abexinostat on A549 and H460 cells in normoxia (O^2^ 21%) [22, 23]. Cells were treated with abexinostat at increasing doses for 24h then exposed to 4Gy irradiation. Expression of acetylated histone H3 was tested 1h and 24h after irradiation by western blot. We found a time and concentration-dependent increased acetylated histone H3 in both investigated cell lines (Figure 1G-1H). Effective histone H3 acetylation could be observed as off 1h of exposure to abexinostat with a remaining effect after a prolonged exposure of 24h (Figure 1G-1H).

### Abexinostat increases apoptosis and irradiation-induced apoptosis

**Figure 2:**
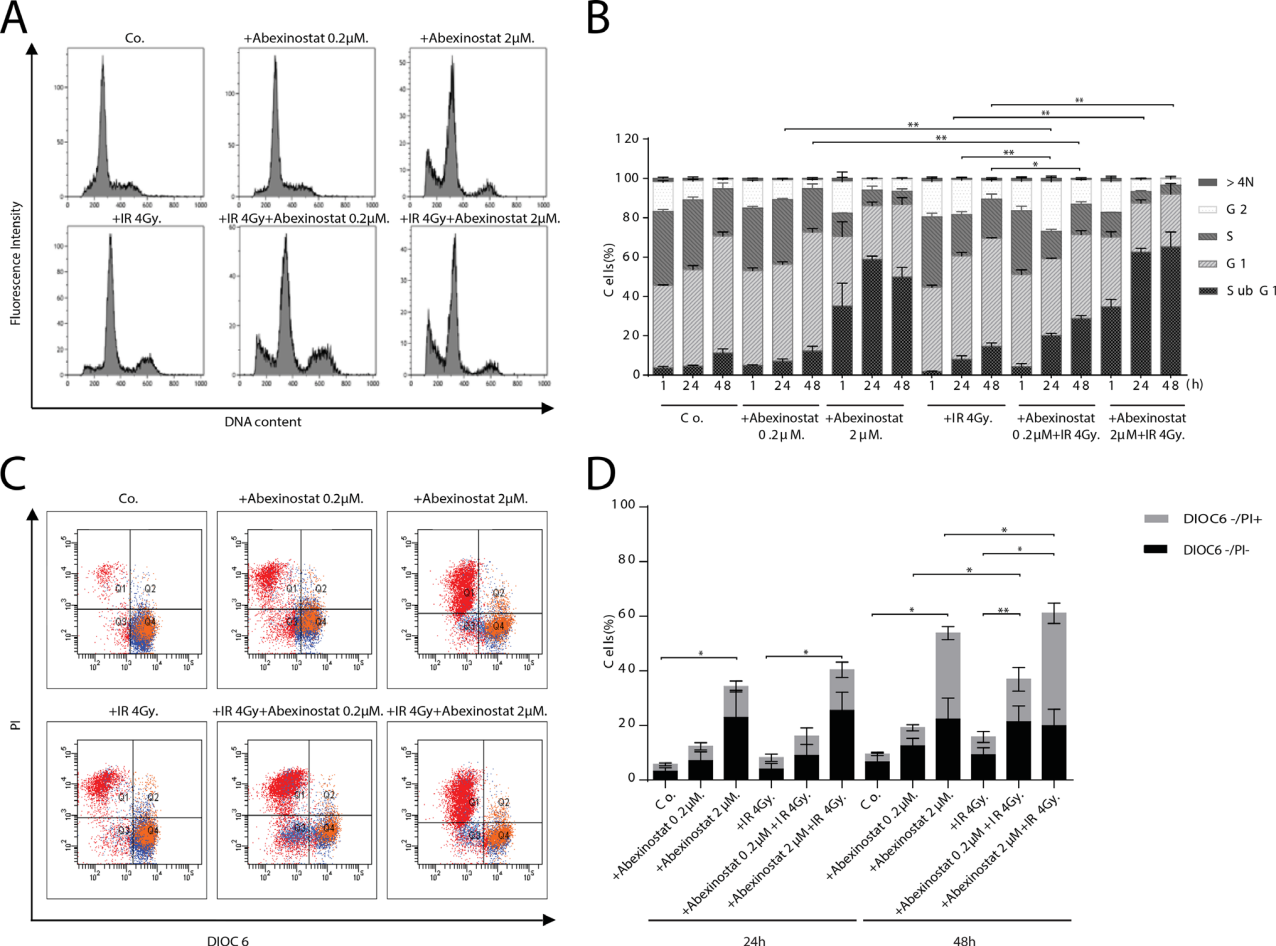
Abexinostat induces H460 apoptosis in a concentration and time-dependent manner H460 cells were treated with indicated increasing concentrations of abexinostat (culture medium as control) for 24h then irradiated or not at 4Gy. Cells distribution in cell cycle phases was counted by flow cytometry assay 1h (B), 24h (B), and 48h after irradiation **A, B.** Apoptosis was assessed at 24h **C, D.** and 48h (C) after irradiation by 3.3′ dihexyloxacarbocyanine iodide (Dioc6) and propidium iodide (PI) staining and flow cytometric analysis (C, D). DIOC6- stands for DIOC6 low staining. Combination of abexinostat and irradiation 4Gy significantly induced subG1 population (A, B) and apoptosis (C, D) (p< 0.05) compared to abexinostat single drug treatment or irradiation alone at 48h. Flow cytometry results (A, C) represent one of three independent experiments. Histograms (B, D) represent means ± SD (n = 3; t-test; *P < 0.05; **P < 0.01). Error bars represent standard deviations.

We found a concentration and time-dependent increased percentage of subG1 population when treating H460 cells with abexinostat, either alone or in combination with irradiation 4Gy. We observed enhanced cell death after exposure to abexinostat 0.2μM for 24 hours before irradiation in H460 cells (Figure 2A-2B). Similar combination yielded to a significant depletion of S-phase populations when H460 cells were exposed to abexinostat 2μM for 24 hours prior irradiation (Figure 2B). In contrast, there was no increased subG1 population nor enhanced cell death observed when starting abexinostat concomitantly with irradiation compared to irradiation alone (data not shown).

To assess whether the observed increase in subG1 population was due to increased apoptosis we used a staining by propidium iodide (PI) and 3.3′ dihexyloxacarbocyanine iodide (DIOC6) which labels mitochondria and correlates to the reduction of mitochondrial trans-membrane potential. Cells were stained with DIOC6 and PI to determine the mitochondrial transmembrane potential and viability, respectively. We monitored the frequency of dying and dead cells by co-staining with the mitochondrial transmembrane potential sensitive dye DiOC6 and the vital dye PI, considering that dying cells have a reduced transmembrane potential but still intact plasma membranes (DiOC6 low/PI-) while dead cells have permeabilized plasma membranes (DiOC6 low/PI+) as described by Zamzami et al [24, 25]. H460 cells were treated with abexinostat at 0.2μM or 2μM for 24h before expose to 4Gy irradiation. PI and DIOC6 staining were performed 24h or 48h after irradiation. Abexinostat significantly increased both basal and post irradiation cell death as shown by increased DIOC6-/PI+ population in H460 cells relative to culture medium controls in a dose dependent manner (Figure 2C-2D). Abexinostat significantly increased both basal and post irradiation DIOC6-/PI- population 48h after irradiation suggesting apoptosis induction with mitochondrial depolarization in H460 cells (Figure 2C-2D).

### Apoptosis induced by abexinostat is caspase-dependent

**Figure 3:**
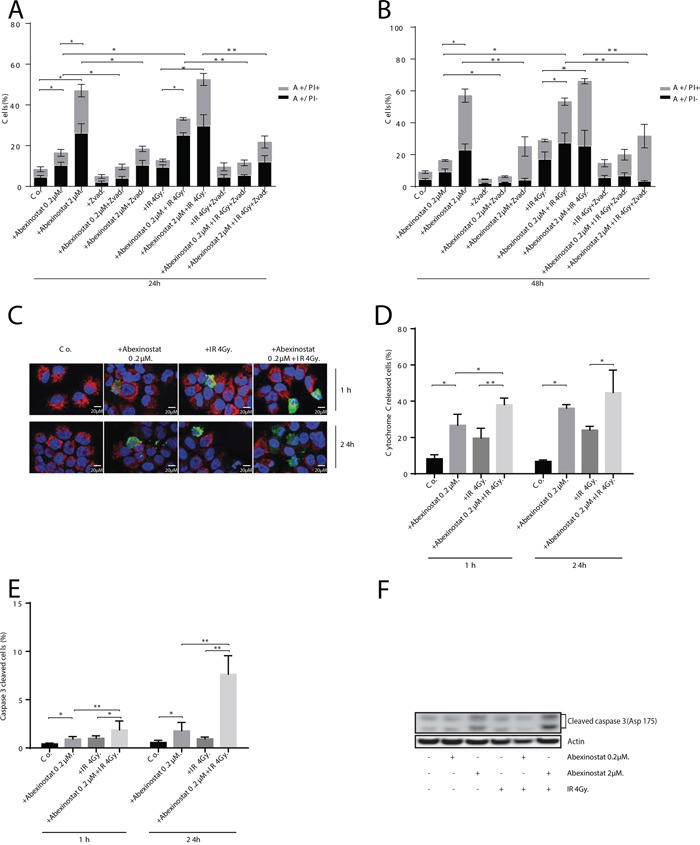
Abexinostat induced apoptosis is mediated by mitochondrial depolarization and caspases activation H460 cells were treated with indicated increasing concentrations of abexinostat (culture medium as control) for 24h then irradiated or not at 4Gy. Apoptosis was assessed 24h **A.** and 48h **B.** after time of irradiation by AnnexineV (A+/−) and propidium iodide (PI+/−) staining with or without Z-Vad used as an apoptosis caspase dependent inhibitor by flow cytometric analysis. Histograms represent means of flow cytometry results ± SD of three independent experiments (n = 3; t-test; *P < 0.05; **P < 0.01). Error bars represent standard deviations. Cytochrome C release and caspase 3 activation were assessed 1h and 24h after time of irradiation by cytochrome C immunostaining used as a mitochondrial depolarization marker **C, D.** and caspase 3 immunostaining used as an apoptosis marker **(C) E.** Positive caspase 3 cells (in green) and cytochrome C released cells (in blurred red) (C) were counted microscopically from 300 cells per condition (D-E). Results represent means ± SD (n = 3; t-test; *P < 0.05; **P < 0.01). Error bars represent standard deviations. Expression of activated caspase 3 in H460 cells treated with indicated increasing concentrations of abexinostat was evaluated by Western blotting 24h after irradiation or not at 4Gy **F.**

To investigate whether observed apoptosis was caspase-dependent, we assessed AnnexinV and PI staining with or without Z-vad used as an apoptosis caspase-dependent inhibitor. H460 cells were treated with indicated increasing concentrations of abexinostat for 24h then irradiated or not at 4Gy. Assessment was performed 24h and 48h after time of irradiation (Figure 3A-3B). Z-vad significantly decreased AnnexinV+/PI- population in H460 cells treated with abexinostat 0.2 and 2μM (Figure 3A-3B). We equally showed that caspase-dependent apoptosis was significantly increased by the abexinostat-IR combination compared to irradiation alone (Figure 3A-3B). Cells treated with abexinostat 0.2μM only showed a moderate increased cell death, whereas cells treated with combined irradiation and abexinostat 0.2μM showed a significant increase in caspase-dependent apoptosis (Figure 3A-3B). In contrast, exposure to higher doses of abexinostat (2μM) alone was associated with a higher proportion of cell death (Annexin V+/PI+). At this dose (2μM) combined irradiation and abexinostat treatment did not showed any significant difference in caspase-dependent apoptosis between abexinostat alone and the combined treatment possibly due to a high cytotoxicity of abexinostat alone as shown by a mean cell death rate of 20.9% and 34.2% at 24h and 48h respectively (Figure 3A-3B). To confirm these results we assessed caspase 3 activation by assessing caspase 3 cleavage and cytochrome C release in H460 cells by immunostaining and found abexinostat 0.2μM significantly increased both basal and post irradiation caspase 3 cleavage and cytochrome C release 1h and 24h after irradiation (Figure 3C-3E). These findings were further supported by increased cleaved caspase 3 expression after abexinostat 2μM either alone or in combination with irradiation as shown by western blotting 24h after irradiation (Figure 3F).

### Abexinostat increases radiation-induced persistent DNA double strand breaks (DSBs)

**Figure 4:**
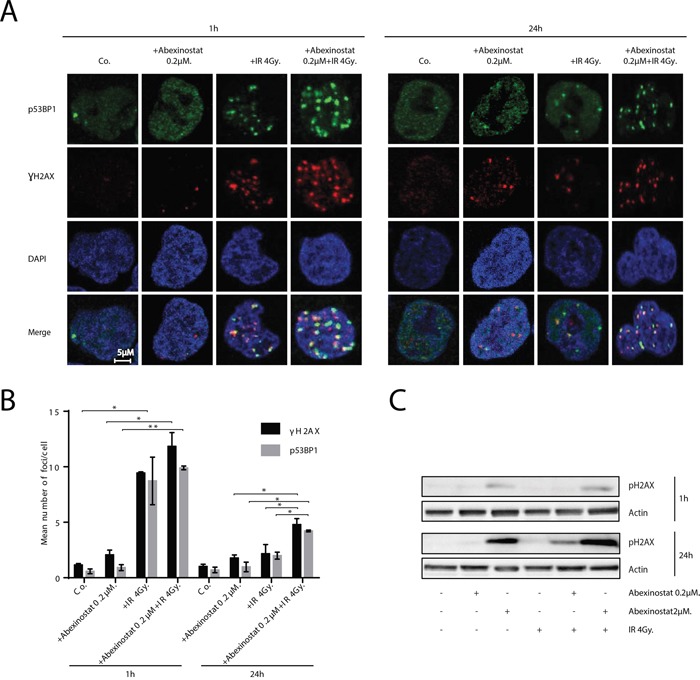
Abexinostat induces DNA-DSBs and prolongs radiation-induced residual DNA damage Immunofluorescence images show representative ƔH2AX/p53BP1-positive nuclear foci (red/green respectively) representing residual DNA-DSBs of 4Gy irradiated H460 cell cultures treated with indicated concentrations of abexinostat **A.** After fixation, cells were stained against ƔH2AX, p53BP1 plus DAPI for nuclei. ƔH2AX/p53BP1-positive foci were counted microscopically from 300 cells per condition **B.** Histograms (B) represent means ± SD (n = 3; t-test; *P < 0.05; **P < 0.01). Error bars represent standard deviations. H460 cells were treated with indicated increasing concentrations of abexinostat (culture medium as control) for 24h prior to irradiation 4Gy **C.** Expression of pH2AX (C) was assessed 1h and 24h after irradiation by Western blotting. Actin served as loading control. Representative Western blots are shown (n=3).

We decided to assess ƔH2AX and p53BP1 foci by immunostaining as indicators of DNA DSBs (Figure 4A-4B). H460 cells were treated with indicated increasing concentrations of abexinostat for 24h, then irradiated at 4Gy. We observed, 1h after irradiation alone, an increase in ƔH2AX and p53BP1 foci number which returns to basal control levels 24h after irradiation (Figure 4A-4B). We found that pretreatment with abexinostat significantly increases ƔH2AX and p53BP1 foci post irradiation in H460 cells 24h after irradiation (Figure 4A-4B). These results indicate that DNA DSBs persist longer after abexinostat and irradiation combination than after irradiation alone in H460 cells. Interestingly an increased rate of persistent DNA DSBs induced by abexinostat and irradiation combination remains 24h after irradiation suggesting a prolonged impairment in the DNA repair capacity (Figure 4A-4B).

The induction of prolonged DNA DSBs by abexinostat was further supported by increased expression of phospho-H2AX induced by abexinostat 2μM in H460 cells as shown by western blotting at 1h after irradiation at 4Gy (Figure 4C). In addition, we observed an increased expression of phospho-H2AX induced by abexinostat 2μM 24h after irradiation at 4Gy which was stronger than at 1h after irradiation corroborating the persistence of DNA DSBs induced by abexinostat and irradiation combination more profoundly than by abexinostat alone (Figure 4C).

### Abexinostat decreases DNA damage signaling and repair

**Figure 5:**
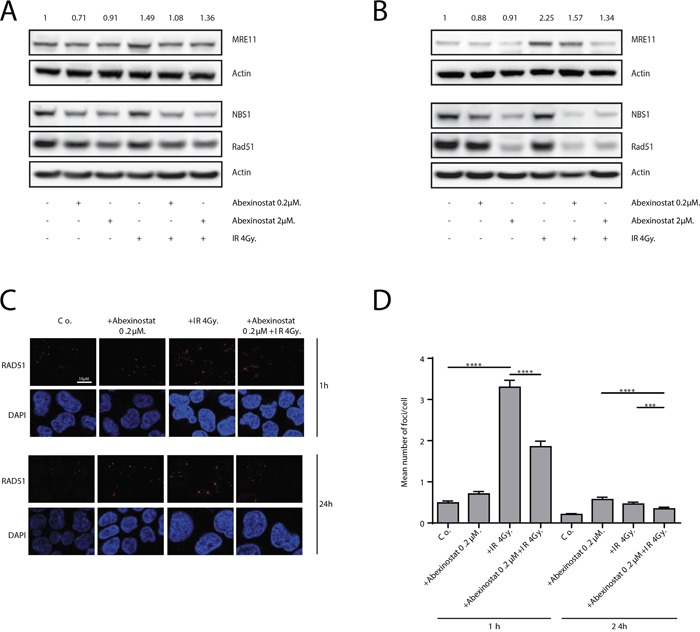
Abexinostat impairs radiation-induced DNA damage repair H460 cells were treated with indicated increasing concentrations of abexinostat (culture medium as control) for 24h prior to irradiation 4Gy. MRE11, NBS1 and Rad51 expression were assessed 1h **A.** and 24h **B.** after irradiation by Western blotting. Actin served as loading control. Representative Western blots are shown (n=3). MRE11 blots were quantified using Image J software and normalized to the control. Immunofluorescence images show representative Rad51-positive nuclear foci (red) representing residual DNA-DSBs of 4Gy irradiated H460 cell cultures **C.** After fixation, cells were stained against Rad51 plus DAPI for nuclei. Rad51-positive foci were counted microscopically from 300 cells per condition **D.** Histograms represent the mean number of foci per cell ± SD (n = 3; t-test; ***P < 0.005; ****P < 0.001). Error bars represent standard deviations.

As persistent increased DSBs might be due to a decreased DNA repair, we tested the hypothesis that abexinostat impairs DNA repair. We therefore evaluated the MRE11/Rad50/NBS1 (MRN) complex which initiates irradiation induced DSBs repair. H460 cells were treated with indicated increasing concentrations of abexinostat for 24h then irradiated at 4Gy. We evaluated the levels of MRE11 and NBS1 proteins by western blotting. We showed a dose-dependent decrease in both protein expression levels, slight at 1h and more marked at 24h after irradiation, as shown by quantification of MRE11/Actin blots normalized to the control (Figure 5A-5B). At 24h abexinostat and irradiation combination markedly decreased levels of MRE11 and NBS1 proteins compared to abexinostat alone (Figure 5B). These results suggest an impaired initial processing of DSBs repair byHR and/orNHEJ which are the two major DNA repair pathway involved in DSBs repair after irradiation. Since Rad51 is one of the key DSBs repair protein in the HR pathway, we decided to further examine its involvement in the observed DNA DSBs persistence. H460 cells were treated with increasing concentrations of abexinostat prior to irradiation. We observed, by western blotting, 1h after irradiation, a moderate decreased level of Rad51 after abexinostat treatment alone. When combining abexinostat with irradiation 4Gy similar results were observed 1h after irradiation (Figure 5A). Interestingly, this decrease was more pronounced 24h after irradiation when cells were treated with abexinostat in combination (Figure 5B). Reduction in Rad51 level was not only time dependent but dose dependent as well (Figure 5B). Exposure to abexinostat 0.2μM combined to irradiation potentiated Rad51 reduction compared to abexinostat alone (Figure 5B). These findings were further supported by an immunofluorescence staining for Rad51 foci (Figure 5C-5D). In contrast after irradiation alone, we observed an increase in Rad51 foci 1h after 4Gy and a return to control level 24h after irradiation. When combining abexinostat with irradiation the increase in RAD51 foci 1h after irradiation was significantly lower than with irradiation alone (Figure 5C-5D). Moreover we observed a prolonged reduction in the number of Rad51 foci 24h after irradiation compared to irradiation alone (Figure 5C-5D).

### Abexinostat potentiates tumor growth delay in combined modality treatments with acceptable toxicity *in vivo*

**Figure 6:**
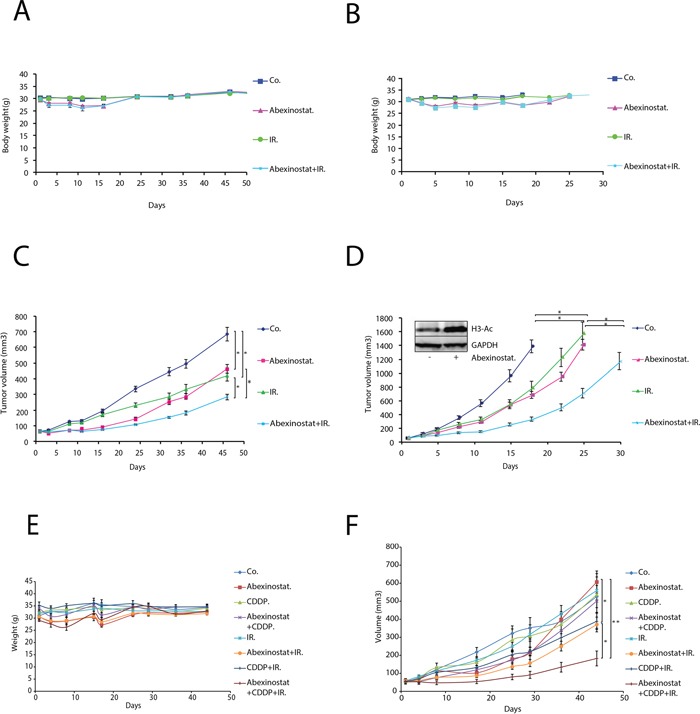
Abexinostat significantly potentiates radio-induced tumor growth delay with acceptable toxicity in A549 and H460 NSCLC xenograft mouse models Mice bearing A549 or H460 xenografts were treated with +/− abexinostat IP 25mg/kg X 2/d (4 days on, 3 days off, 4 days on, 3 days off, 4 days on) and +/− irradiation at 2Gy/d on day 2, 3 and 4. Vehicle (HβCD 30%) was used as control. Body weight was measured twice weekly for mice bearing A549 or H460 xenografted tumors (**A, B.** respectively). Growth delay of abexinostat treated mice bearing A549 **C.** or H460 **D.** xenografts were measured as described under materials and methods. Results represent means ± SD (n = 2; 10 mice per group; t-test; *P < 0.05; **P < 0.01). Acetylation of histone H3 was evaluated after 4 consecutive days of abexinostat IP 25mg/kg X 2/d by Western blotting in *ex vivo* H460 tumors 4h after the second IP (D). GAPDH served as loading control. Representative Western blots are shown (n=3). Mice bearing A549 xenografted tumors were treated with +/− abexinostat IP 25mg/kg X 2/d (5 days on, 4 days off, 3 days on, 3 days off, 5 days on) +/− Cisplatin IP 1mg/kg 1h before time of irradiation on day 2 and day 16 and +/− irradiation at 2Gy/d on day 2, 3 and 4. DMSO was used as control. Body weight was measured twice weekly for mice bearing A549 xenografted tumors **E.** Growth delay of mice bearing A549 xenografts were measured **F.** as described under materials and methods. Results represent means ± SD (10 mice per group; t-test; *P < 0.05).

We next further evaluated the effects of abexinostat on response to irradiation *in vivo*. We assessed tumor growth of A549 or H460 mice xenografts treated with abexinostat and/or irradiation as described under material and methods and in Supplementary Figure 2A. Body weight was used as an indicator of mice health status (Figure 6A-6B for mice with A549 and H460 xenografts respectively). As shown in Figure 6A and 6B, we observed a maximum mean body weight loss in treated groups of 12.4% and 8.7% on day 11 in abexinostat combined with irradiation and abexinostat alone treated groups respectively. This weight loss did completely recover after treatment (on day 24) for all treated groups and therefore was considered acceptable. Abexinostat treatment alone resulted in significant inhibition of tumor growth in both A549 (p=0.005) (Figure 6C) and H460 (p=0.0009) (Figure 6D) xenografts in the same range as irradiation alone. Interestingly, tumor growth, in both models, was significantly delayed by the combination of abexinostat and irradiation compared to a single treatment modality by abexinostat or irradiation alone. For A549, we observed a mean tumor volume of 464.54 mm^3^ (SEM= 28.02) versus 283.78 mm^3^ (SEM= 19.71) at day 46 after starting treatment in abexinostat alone and combination treated groups respectively (p=0.006) (Figure 6C). For H460, tumor growth was much faster than A549 with a mean tumor volume of 688.13 mm^3^ (SEM= 52.98) versus 331.77 mm^3^ (SEM= 31.24) at day 18 after starting treatment in the abexinostat alone and in the combination treated groups respectively (p=0.034) (Figure 6D). To confirm drug diffusion in the tumor, abexinostat concentration was measured *ex vivo* from tumor homogenates after 4 consecutive days of abexinostat (25 mg/kg BID) treatment. Mean abexinostat concentrations in the tumor were 477ng/mL, 428ng/mL and 73.4ng/mL at 4h, 7h and 20h respectively after the second IP of the day in the abexinostat alone group. To assess HDAC inhibitory efficiency *in vivo* of abexinostat we evaluated the acetylation of histone H3 by western blot using *ex vivo* homogenates from H460 tumors extracted after exposure to abexinostat. In agreement with *in vitro* studies, we observed increased acetylated histone H3 in abexinostat treated group compared to control after 4 consecutive days of abexinostat (Figure 6D). Increased acetylated histone H3 correlated with the antitumor effect of abexinostat as shown by the observed tumor growth delay in Figure 6D. Data have been expressed in Kaplan Meier survival curves (Supplementary Figure 2B-2C). It is of note that mice have been sacrificed after a statistically significant volume difference was reached. Therefore a number of data were sensored and we couldn't observe a statistically significant difference in overall survival.

As the standard of care for locally advanced NSCLC in clinic is based on concomitant radio chemotherapy with a platinum based drug doublet, we decided to investigate the triple combination of abexinostat with irradiation and cisplatin (CDDP). We hypothesized that the limited toxicity of abexinostat and irradiation combination might leave room for adding CDDP and that this addition might potentiate the effect of CDDP combined to irradiation. We assessed tumor growth of A549 xenografts treated with abexinostat and/or CDDP and/or irradiation as described under material and methods and in Supplementary Figure 2D. Body weight was measured twice weekly. Treatment schedule was adapted to tolerance and toxicity profile. As shown in Figure 6E we observed a maximum mean body weight loss in treated groups of 10.7% and 6.9% on day 17 for abexinostat alone and abexinostat combined with irradiation respectively and 9.43% on day 6 for the triple combination of abexinostat, irradiation and CDDP with a marked effect after each CDDP injection delivered on days 2 and 16. This weight loss did completely recover after treatment for all groups on day 25. Abexinostat combined with irradiation exhibited significant tumor growth delay as compared to the abexinostat alone group (p= 0.046). Interestingly the triple combination of abexinostat, CDDP and irradiation exhibited a border line significant tumor growth delay as compared to the double combination of abexinostat and irradiation (p= 0.05). Significant growth delay was observed as well with the triple combination compared to the combination of CDDP and irradiation (p= 0.03) or compared to abexinostat alone (p= 0.004) (Figure 6F). Survival data have been expressed in Kaplan Meier survival curves (Supplementary Figure 2E). It is of note that in the triple combination with CDDP, mice have been sacrificed after a statistically significant volume difference was reached between above indicated groups on day 51 and 65. Therefore a number of data were sensored and we couldn't observe a statistically significant difference in overall survival. Nevertheless a Logrank test for trend was performed and showed a p= 0.0096 suggesting that with a longer follow up we could see a difference in survival.

## DISCUSSION

Abundant pre-clinical studies and early clinical trials have shown a promising anti-tumor effect of HDACi alone or in combination with chemotherapy agents linked with an induced alteration in DNA repair capacity [26–28]. More recently, rapidly expanding pre-clinical *in vitro* and *in vivo* data have shown a radiosensitizing effect of various HDACi in different tumor cells explained by changes is chromatin conformation or reduced DNA repair capacity when combined with irradiation [3]. *In vivo* studies have suggested such radiosensitizing effect in various tumor types [22, 28, 29, 30–32, 33, 34, 35, 36, 28, 37, 38–42, 43]. Since HDACi effects seem to be dependent on both the type of tumor cell and the specific HDACi used [44, 45] further preclinical studies are needed to identify the best combinations to increase chances of success in clinical trials, particularly if HDACi are used in combination with radiotherapy. By testing *in vitro* and *in vivo* abexinostat, a recently developed hydroxamic acid–based pan-HDACi currently being evaluated in combination with radiotherapy in a phase I clinical trial in metastatic patients, we add further data supporting the rational for the combination of HDACi and radiotherapy namely in NSCLC in future clinical trials.

Our results underscore for the first time the importance of the administration schedule when combining abexinostat and irradiation. We demonstrated with abexinostat that administration 24 hours before irradiation induces a radiosensitizing effect *in vitro* and potentiates tumor growth delay *in vivo*. This effect, which was not observed when abexinostat was started concomitantly with irradiation (data not shown), show consistency with a mechanism that does require time for epigenetic changes as suggested with some HDACi so far including LBH589, vorinostat, TSA and NDACI054 [23, 28, 29, 46]. These results are of importance for clinical translation and justify starting abexinostat before irradiation in the ongoing and future combination trials.

Abexinostat schedule given BID based on days on and off treatment instead off a continuous treatment was chosen for this study based on pharmacokinetic and pharmacodynamic data, tolerability and activity of abexinostat observed in the clinical study PCYC-0402 and in our phase I study combining abexinostat and radiotherapy currently under publication [47, 48]. Starting from the dose of 12.5mg/Kg BID we have tested increasing doses following the published schedule [48]. We have shown that 25mg/Kg BID was still well tolerated and therefore have chosen this dose and schedule.

For *in vivo* experiments, we have selected a fractionated schedule with 2Gy per fraction to keep the dose per fraction similar to what is used in normofractionated schedules in the clinic for the treatment of non-operable locally advanced NSCLC. The total dose was kept relatively low (6Gy) to better see the effect of the combination and show a proof of concept.

Hypoxic tumor cells present a more aggressive phenotype often associated with lower radio and chemo-sensitivity. It is of high importance to evaluate the efficacy of potential radiosensitizers under normoxia and hypoxia since human tumors including NSCLC often contain a substantial fraction of hypoxic cells. Saelen *et al* have shown *in vitro* and *in vivo* that vorinostat enhances radiosentivity of colon cancer cells almost counter-balancing hypoxia-induced radioresistance [32]. The ability of HDACi to overcome hypoxia-related radioresistance would be a major advantage for the treatment of hypoxic tumors such as NSCLC.

Although most HDACi have showed some cytotoxic effect and cell death induction capacities through apoptosis, necrosis or autophagy, the precise molecular mechanism underlying the balance between the different cell death mechanisms according to tumor types and HDACi specificities remains unknown [1]. Many questions still remain regarding the molecular mechanisms of radiosensitization by abexinostat and HDACi. However, it is possible that the capacity of abexinostat to induce double strand breaks, while reducing the capacity to repair damaged DNA, might not only have an important role per se but also by increasing irradiation-induced apoptosis. This provides a molecular rationale for the synergistic activities of HDACi and irradiation. Even though the main cell death mechanism induced by irradiation is mitotic cell death, potentiating other cell death pathways such as apoptosis might end in an increased antitumor efficacy supporting the clinical interest of the combination. [1, 28] Previous studies have shown either a pro-apoptotic effect or no impact on apoptosis depending on the type of HDACi used and the type of cell [23, 28, 49]. In our study, we investigated the effects of abexinostat on cell proliferation, cell cycle progression and apoptosis in both A549 and H460 NSCLCC (data partially shown, Figures 1-3). In H460 cells, treatment with abexinostat induced an increased sub G1 population corresponding to increased apoptosis, as shown by AnnexinV/propidium iodide flow cytometry, without significant changes in cell-cycle profile (Figure 3A-3B). The apoptotic response induced by abexinostat is caspase dependent as shown by the increased level of cleaved caspase 3 and a significant reduction of induced apoptosis when using Z-VAD, a pan-caspase inhibitor (Figure 3A-3B, 3F) [1, 50]. We have additionally found that abexinostat apoptotic response involves and potentiates the “intrinsic” mitochondrial apoptosis pathway as shown by reduced DIOC6 uptake upon the drop of mitochondrial transmembrane potential and increased cytochrome C release (Figures 2-3) [1, 51]. Our findings suggest that abexinostat renders H460 cells more susceptible to apoptosis after irradiation and that the “intrinsic” apoptosis pathway is one mechanism through which abexinostat and irradiation interacts.

Response to irradiation is recognized to be driven by the repair efficiency of radiation induced DNA damage in which double strand breaks play a major role [52]. Mounting reports suggest that HDACi exert a radiosensitizing effect by down regulating DNA repair pathways in various tumor types with different HDACi [9]. It has previously been shown, *in vitro*, that abexinostat (also known as PCI-24781 or S78454) can down regulate HR in HCT116 cells by repressing transcription and consequently expression of Rad51 which might explain radiosensitization induced by abexinostat [18]. Our results confirm in H460 cells, which are less radiosensitive than HCT116 cells, that the radiosentizing effect of abexinostat is correlated with a decreased level of RAD51 protein and RAD51 foci (Figure 5). This work provides contributive evidence that HDACi can play an important role in HR modulation by regulating not only effectors level but formation of DNA repair complexes as well considered to represent the “repairosome” [10]. A second approach has been used to asses DNA repair by evaluating the expression of phosphorylated H2AX, γH2AX and 53BP1 foci formation. H2AX is rapidly phosphorylated at sites of DNA double strand breaks and acts with 53BP1 as a damage signaling protein forming nuclear foci visible by immunostaining on fluorescence microscopy [9, 53]. The exact mechanism of action of these proteins remain unclear, but the variation in the number of such foci is thought to be representative of the induction and repair of DNA double strand brakes [10]. Our findings suggest not only a decreased repair with prolonged expression of γH2AX and 53BP1 foci (Figure 4) in great similarity with most previously published data on radiosensitization by HDACi, but also an associated increased DNA damage induction with increased double strand brakes, as shown by the increased number of γH2AX and 53BP1 foci as early as 1 hour after irradiation.

Lung cancer is still the leading cause of cancer death. Radio-chemotherapy remains the standard of care for locoregional unresectable lung tumors [20]. Despite strong rational and promising results from *in vitro* and *in vivo* studies, the use of targeted therapeutic agents combined with radiotherapy in NSCLC is still limited. Several attempts have been made to overcome the drug-radiotherapy combination challenge in early clinical trials. Recently, most of these studies have been focusing on the use of anti-EGFR and other signal transduction inhibitors. Due to their role in diverse oncogenic pathways a lack of success of these agents in unselected patients in the clinic was observed. Fearing possible toxicities of a triple combination the RTOG 1306 phase II randomized trial is currently assessing the impact on progression free survival of these targeted agents (either erlotinib for EGFR mutated tumors or crizotinib for ALK translocated tumors) before concurrent chemoradiotherapy in patients with stage III Non-small Cell Lung Cancer (ClinicalTrials.gov identifier: NCT01822496) but the question of concomitant triple combination deserves cautious assessment and could open the way for new improvements. Evidence linking radiosensitivity and epigenetics are still scarce and mostly based on *in vitro* data [54]. Synergistic effects of anti-EGRF and HDACi have been reported in NSCLC by Edwards *et al* who showed that the HDACi LBH589 induces apoptosis only in EGFR mutated NSCLC [55]. Controversially in our findings, we demonstrate that the potentiating effect of abexinostat and irradiation with apoptosis induction is observed in H460, an EGFR wild type cell line.

Few *in vitro* and *in vivo* studies have shown synergistic anti-tumor effect between CDDP and HDACi [48, 56]. The standard of care for locally advanced unresectable NSCLC patients is based on concomitant radio chemotherapy with a platinum based drug doublet [20]. Various platinum based doublets have been tested with concomitant radiotherapy over the last years with limited improvement in overall survival when comparing one doublet to another [57]. Our findings show for the first time an *in vivo* potentiation of the anti-tumor effect of the CDDP-radiotherapy combination by an HDACi. Abexinostat increases tumor growth delay when used in triple combination in a borderline significant way (p=0.05) with a clear trend to improved survival (logrank test for trend p= 0.0096) (Figure 6F, Supplementary Figure 2E). Given that Abexinostat can induce DNA double strand breaks and lower the capacity to repair damaged DNA and that cisplatin and irradiation cause DNA damage, combining abexinostat, irradiation and cisplatin might end up in an increased tumor cell apoptosis. Abexinostat might therefore potentiate the antitumor effect of cisplatin and irradiation. This should serve as the basis for further investigations and potential clinical translation to address the crucial need for improvement in NSCLC treatment.

Concerns about toxicities and feasibility of the triple combination of abexinostat, irradiation and cisplatin might be raised due to the risk of hematologic toxicity of both cisplatin and abexinostat (thrombocytopenia and leukopenia). Hematologic toxicity of irradiation is limited and mostly due to the irradiated bone marrow volume. Given that abexinostat can be administered clinically with minimal toxicity at recommended dose and that the risk of induced thrombocytopenia is reduced by using clinically optimized treatment schedule, even in combination with radiotherapy as shown in our phase I trial under publication, concerns regarding the triple combination are limited but will justify a careful hematologic follow up [47, 48]. Other cisplatin toxicities such as.nephro- and neurotoxicity should not be at increased risk given that neither abexinostat nor other HDACi seem to increase this risk. Nevertheless the toxicity profile of each treatment of this combination suggest a possible risk of increased nausea specially if the irradiated volume includes part of the gastrointestinal track which should be carefully assessed in the clinic even though no such toxicity was observed *in vivo*.

Altogether, our data demonstrate *in vitro* and *in vivo* a potentiation of anti-tumor effect of irradiation by abexinostat in NSCLC models. Moreover, our work suggest for the first time to our knowledge promising triple combination opportunities with HDACi, irradiation and CDDP which deserves further investigations and could be of major interest in the treatment of NSCLC where improvement in efficacy is crucially needed but limited by the toxicity of current combined treatments.

## MATERIALS AND METHODS

### Cell lines, cell culture, and irradiation

A549 and H460 human non-small cell lung carcinoma (NSCLC) cell lines were obtained from ATCC (Manassas, VA, USA). A549 cells were cultured in F-12K Medium (ATCC, Manassas, VA, USA) supplemented with 10% fetal bovine serum (FBS) (PAA laboratories) and 100 units/mL penicillin G sodium, 1% Hepes and 1% Sodium/pyruvate (Life Technologies, Carlsbad, CA, USA). H460 cells were cultured in RPMI Medium (Life Technologies, Carlsbad, CA, USA) supplemented as previously described for F-12K Medium. All cells were cultured at 37°C in a humidified atmosphere containing 5% CO2 either in normoxia (21% O^2^) or in hypoxia (0.1% O^2^). For hypoxia conditions pre-plated cells were incubated with 0.1% O^2^ for 24h then treated with abexinostat at indicated dose for 24h and irradiated at indicated dose in hypoxia. Treatment by abexinostat in hypoxia (0.1% O^2^) was maintained according to indicated time. Irradiation was delivered at room temperature using single doses (2–6Gy) on an IBL-687 irradiator (CIS-Bio International) with a dose rate of 1Gy/min.

### Chemicals and antibodies

Abexinostat (Code: S78454-1; also coded PCI-24781-HCI by Pharmacyclics) provided by Technologie Servier (Orléans, France) was solubilized at 10mM in DMSO aliquoted and stored at −20°C. The frozen stock was thawed only once. Intermediate and final dilutions were prepared extemporaneously in complete culture medium for *in vitro* experiments and 2-hydroxypropyl-beta-cyclodextrin (HβCD) 30% for *in vivo* experiments protected from daylight. Cisplatin (Cis-diammine-platinum (II) dichloride or CDDP) was purchased from Sigma Aldrich St. Quentin Fallavier, France). CDDP was diluted in HβCD 30% aliquoted and stored at −20°C. Primary antibodies for detection of Acetyl Histone H3 (Lys9), Cleaved caspase 3 (Asp175), MRE11, p95/NBS1 were purchased from Cell Signaling Technology. Primary antibodies against phospho H2AX and GAPDH were obtained from Millipore. Primary antibodies against cytochrome C, 53BP1, Rad51 and β-actin were respectively purchased from BD pharmingen, Bethyl Laboratories, Calbiochem and Sigma. Horseradish peroxidase-conjugated goat anti mouse or anti rabbit secondary antibodies were from Southern Biotechnology. Z-Val-Ala-Dl-Asp-fluoromethylketone (Z-VAD-FMK) was purchased from Bachem and 2-Hydroxypropyl-β-cyclodextrin (HβCD) from Sigma. The solvent DMSO or culture medium was used as control. Cells were treated with abexinostat at indicated concentrations for indicated time period and 24h prior to irradiation unless specified otherwise.

### WST-1 assay

Cells were seeded in 96-well plates in growth medium overnight. Cells were treated with vehicle (DMSO) or different concentrations of abexinostat for 48h, then incubated with WST-1 reagent at 37°C for 3h. After incubation, superoxide produced by living cells reduces the tetrazolium salt, WST-1 to produce a soluble formazan. The absorbance was measured by fluorimetry at 450nm. Values were normalized to a non-treated control. IC50 values represent abexinostat concentration reducing by 50% the number of viable cells.

### Total protein extracts and western blotting

Western blotting was performed to detect Acetyl Histone H3 (Lys9) (H3-Ac), Cleaved caspase 3 (Asp175), phospho H2AX (pH2AX), MRE11, p95/NBS1 and Rad51. Cells were treated 16 hours after plating in complete medium (containing FBS) at indicated doses and time period with +/− abexinostat +/− irradiation. Total cellular proteins were extracted in lysis buffer: 20mM Hepes, 10mM KCl, 1mM EDTA, 0.1% NP40, 10% glycerol, protease inhibitor cocktail (EDTA-free protease inhibitor tablets, Roche) and phospatase inhibitor (phosphoSTOP tablets, Roche). Protein content was evaluated using a Bio-Rad^®^ kit, and 10μg of protein sample were loaded on a denaturing acryl-amide NuPage Bis-Tris gel (Invitrogen). Following denaturing at 35°C for 5min, 30μg of proteins were loaded on NuPAGE Novex Bis-TRis 4-12% pre-cast gels (Life technologies) and electrotransferred to PDVF membranes (Amersham). Red Ponceau dye was used for protein detection during western blotting and confirmed the quality of protein transfer. After blocking unspecific binding sites in 0.1% Tween -20 (v/v in TBS) with 5% bovine serum albumin, membranes were incubated overnight with primary antibody at room temperature. Primary antibodies were detected with appropriate horseradish peroxidase-conjugated secondary antibodies and were revealed with enhanced Dura detection system (Thermo Fisher Scientific) on a G-Box Chemi XL 1.4 (Syngene) for image recording. Equal lane loading was verified by probing membranes with antibodies specific for β-actin or GAPDH. For *ex vivo* tumor homogenates total cellular proteins were extracted in lysis buffer and western blotting was performed to detect Acetyl Histone H3 (Lys9) (H3-Ac) following the same procedure.

Our blots were quantified using ImageJ software.

### Clonogenic survival assay

A549 and H460 cells were seeded in 4mL culture medium/T-25 flasks with 100 to 3000 cells/flask, so as to yield 10-200 colonies/flask. After 4 hours, cells were treated with indicated concentrations of abexinostat or vehicle as control for 24h followed by irradiation at indicated dose. Medium was changed 24h after irradiation and cells were incubated for 10-14 days in a 37°C, 5% CO2, 21% or 0.1% O^2^ incubator. Cells were then stained with a solution containing crystal violet and ethanol for 15min and individual colonies (>50 cells) were counted. Data from treated cells were normalized against the unirradiated cells (scored as 100% colony forming ability). Plating efficiencies (PE) were calculated as follows: numbers of colonies formed/numbers of cells plated. Surviving fractions (SF) were calculated by dividing the PE of the treated cells by the PE of the controls (unirradiated cells). The radiation dose at 10% survival was calculated by transforming the linear quadratic equation (SF = exp [−αxD-βD^2^]). Sensitization enhancement ratios (SER) were calculated by dividing the radiation dose at 10% cell survival treated with culture medium by the radiation dose at 10% cell survival treated with indicated concentration of abexinostat. Each point on survival curves represents the mean surviving fraction from at least three independent experiments. Experimental data were fitted to the classical linear-quadratic equation (Ln (S) = -αD-βD^2^) through nonlinear least-square regression using Kaleidagraph software (Synergy Software, Reading, PA). We used: Ln (S) = -αD-βD^2^ where S is the survival fraction, D the dose, α and β adjustable parameters depending on the cell line and the treatment used. For each experiment, the mean D10 dose (dose to achieve 10% survival) was recalculated using the mean values of α and β determined from the curves drawn for best fit to the experimental data. In each case, the correlation coefficient was more than 0.99.

### Isobolograms

The cytotoxic interactions of abexinostat and radiation at 10% survival iso-effect (IC90) were evaluated using the isobologram method of Steel and Peckham [58] The isobologram analysis for A549 (G,I) and H460 (H,J) were shown in modified Supplementary Figure 1 in normoxic (G,H) and hypoxic (I,J) conditions. The envelope of additivity, surrounded by Mode I (circles) and Mode II (squares) isobologram lines was constructed from the doses-response curves of abexinostat alone and radiation alone. For each condition the experimental data giving 10% survival was plotted on the graph. A non-parametric Wilcoxon signed rank statistical test was done between experimental data and the predicted minimum data.

### Flow cytometric assay: cell cycle analysis and cell death assay

For analysis of cell cycle sham control and 4Gy-irradiated cells with indicated concentration of abexinostat exposure 24h prior to irradiation were harvested at different time points after irradiation (1- 24- 48 h) and fixed in 70% ethanol. DNA content was stained in propidium iodide (PI) solution (0.1mg/ml PI, 1mg/ml RNase and 20mM EDTA in PBS (pH7.8)). To study cell death, cells were harvested without fixation. H460 cells were stained 24 and 48h after irradiation with 3.3 dihexyloxacarbocyanine iodide (DiOC6 (3) Molecular Probes) and PI. H460 cells were equally stained at same time points with fluorescein isothiocyanate (FITC)-conjugated AnnexinV (MACS, Myltenyi Biotec) +/− Z-VAD-FMK as per instructions of the manufacter. Flow cytometry analysis was performed on a BD LSR II system (BD Biosciences, Stockholm, Sweden). Data were analyzed using BD FACSDiva Software (Becton Dickinson) and FlowJo Software (Verity software House, Topsham, ME, USA). Culture medium was used as a control.

### Immunofluorescence staining

H460 cells were grown on 6-well chamber slides and treated with vehicle (culture medium as a control) or different concentrations of abexinostat for 24h prior to irradiation 4Gy. At the indicated time (1 - 24h) after +/− 4Gy, cells were fixed in 4% paraformaldehyde for 20 minutes and permeabilized in 0.1% SDS for 10 minutes. Unspecific binding sites were blocked with 10% BSA for 30 minutes then slides were incubated with specific caspase 3, cytochrome C, ƔH2AX, 53BP1 or Rad51 primary antibodies (1/200). The slides were washed 3 times in 1X PBS and incubated with Alexa Fluor 488 or Alexa Fluor 568-conjugated species-specific secondary antibodies (Molecular Probes) for 1h (1/500). Cells were counterstained with Hoescht 33342 (Life technologies) and analyzed by fluorescent confocal microscopy on a Leica SP8 using a 63x objective.

### *In vivo* xenograft studies

Animals were maintained in appropriate pathogen-free conditions and experiments followed the Federation of European Laboratory Animal Science Association (FELASA) guidelines. Animal experiments were approved by the local Ethics Committee (CEEA IRCIV / IGR, registered with the French Ministry of research) and were in compliance with the EU 63/2010 directive. Mice were housed under standard conditions (12h light/12h dark at 21∼23°C and 60∼85% humidity) with ad libitum access to sterilized food and water. Female athymic nude mice (Janvier, Le Genest-Gaint-Isle, France) 6-8 weeks of age were inoculated subcutaneously with 2.5.10^6^ A549 or H460 cells newly purchased from ATCC. Cells were tested for mycoplasma contamination and presented none. When tumor size reached 65-100 mm^3^, mice were randomly allocated to four groups (n=10 per group) and treated by intraperitoneal injection (IP) with vehicle (HβCD 30% ), abexinostat 25 mg/kg BID (twice daily) (4days on, 3days off, 4days on, 3days off, 4 days on), vehicle (HβCD 30%) and irradiation at 2Gy/d on day 2, 3 and 4, or combined treatment with irradiation delivered 1h after the second injection of the corresponding day of treatment (Figure 6A-6D, Supplementary Figure 2A). In two independent experiments H460 tumor homogenates were prepared *ex vivo* collecting mice tumors 4h, 7h, 20h after the second abexinostat injection of the day after 4 consecutive days of abexinostat IP 25mg/kg X 2/d. Tumors were immediately frozen with liquid nitrogen after wet weight was recorded. Tumor tissue homogenates were stored at −80°C prior to sample analysis. The final concentration of abexinostat was determined in the tumor by normalization based upon the weight of mouse tumor tissue collected. In an independent experiment following the same procedure, when A549 tumor size reached 65-100 mm^3^, mice were randomly allocated to 8 groups (n=10 per group) and treated with +/− abexinostat IP 25mg/kg X 2/d (5 days on, 4 days off, 3 days on, 3 days off, 5 days on) +/− cisplatin IP 1mg/kg 1h before time of irradiation on day 2 and day 16 and +/− irradiation at 2Gy/d on day 2, 3 and 4, 1h after the second injection of abexinostat of the corresponding day of treatment (Figure 6E-6F, Supplementary Figure 2D). Vehicle (HβCD 30%) was used as control. In all experiments, tumor volumes were calculated using caliper measurements twice a week as follows: volume (mm3) = (length × width^2^)/2. Body weights were measured twice a week. Selective tumor irradiation was delivered using an X-ray tube (Tube Varian NDI 226, 0.87 Gy/min) under a tension of 200KV at 15mA, with a 0.2mm cooper filter and a skin source distance of 21cm. Mice with weight losses greater than 20% or tumor over 1800mm^3^ were sacrificed.

### Statistical analysis

Unless stated differently statistical analyses were performed using GraphPad Prism 6.0 software (GraphPad, San Diego CA). Data are expressed as mean plus or minus standard deviation (SD) or standard error of the mean (SEM) of at least 3 independent experiments. For *in vitro* experiments statistical comparisons were made using an unpaired t test. A two way ANOVA analysis was used to determine significant differences in mean tumor volumes *in vivo*. P values less than 0.05 were assigned significance. Irradiation survival curves were fitted according to the linear-quadratic model.

## SUPPLEMENTARY FIGURES AND TABLE


