# Supplementary material for: Time dependent modulation of tumor radiosensitivity by a pan HDAC inhibitor: abexinostat
Source: Oncotarget. 2017 Jan 25;8(34):56210–27. doi: 10.18632/oncotarget.14813 (PMC5593556; doi:10.18632/oncotarget.14813)
Supplement: Supplementary file 1 [file oncotarget-08-56210-s001.pdf]

Time dependent modulation of tumor radiosensitivity by a pan HDAC inhibitor: abexinostat

SUPPLEMENTARY FIGURES AND TABLE

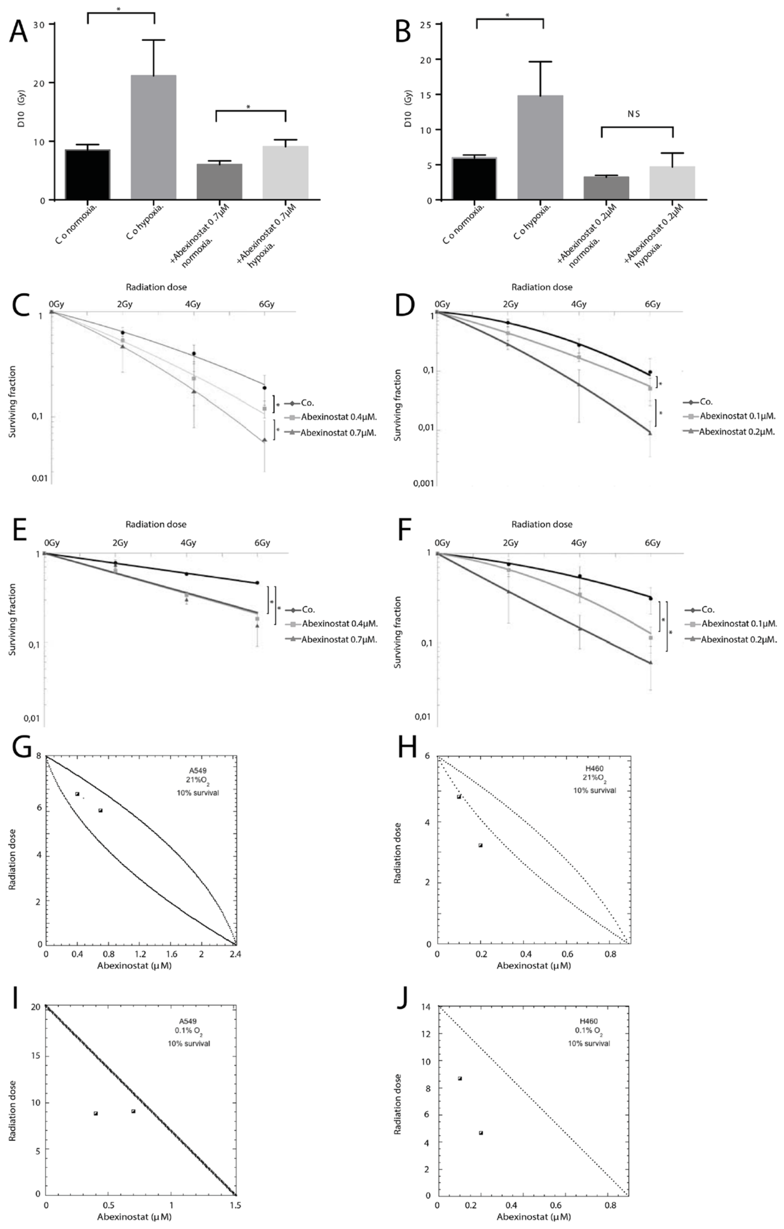

Supplementary Figure 1: Additive and supra-additive effect of irradiation combined with abexinostat *in vitro* in normoxic and hypoxic conditions in NSCLC cells. Doses of radiotherapy in gray (Gy) to achieve 10% cell survival (D10) determined by clonogenic survival assay were lower when adding abexinostat for both A549 **A.** and H460 **B.** respectively at indicated doses. Results represent means ± SD (n=3; t-test; $*P < 0.05$, $**P < 0.01$, NS: non-significant). A549 cells **C, E, G, I.** and H460 cells **D, F, H, J.** were treated with indicated increasing concentrations of abexinostat (culture medium as control) for 24 hours then irradiated or not at indicated doses. Clonogenic radiation survival of abexinostat treated A549 (C, E) and H460 (D, F) cells in normoxic (C, D) or hypoxic (0.1% O^2) (E, F) conditions were measured as described under materials and methods and fit to a linear quadratic model (C-F). Culture medium was used as control. Results represent means ± SD (n=3; t-test; $*P < 0.05$). Error bars represent standard deviations. A supra-additive effect was confirmed by an isobologram analysis for both A549 and H460 cell lines in hypoxia (I-J).

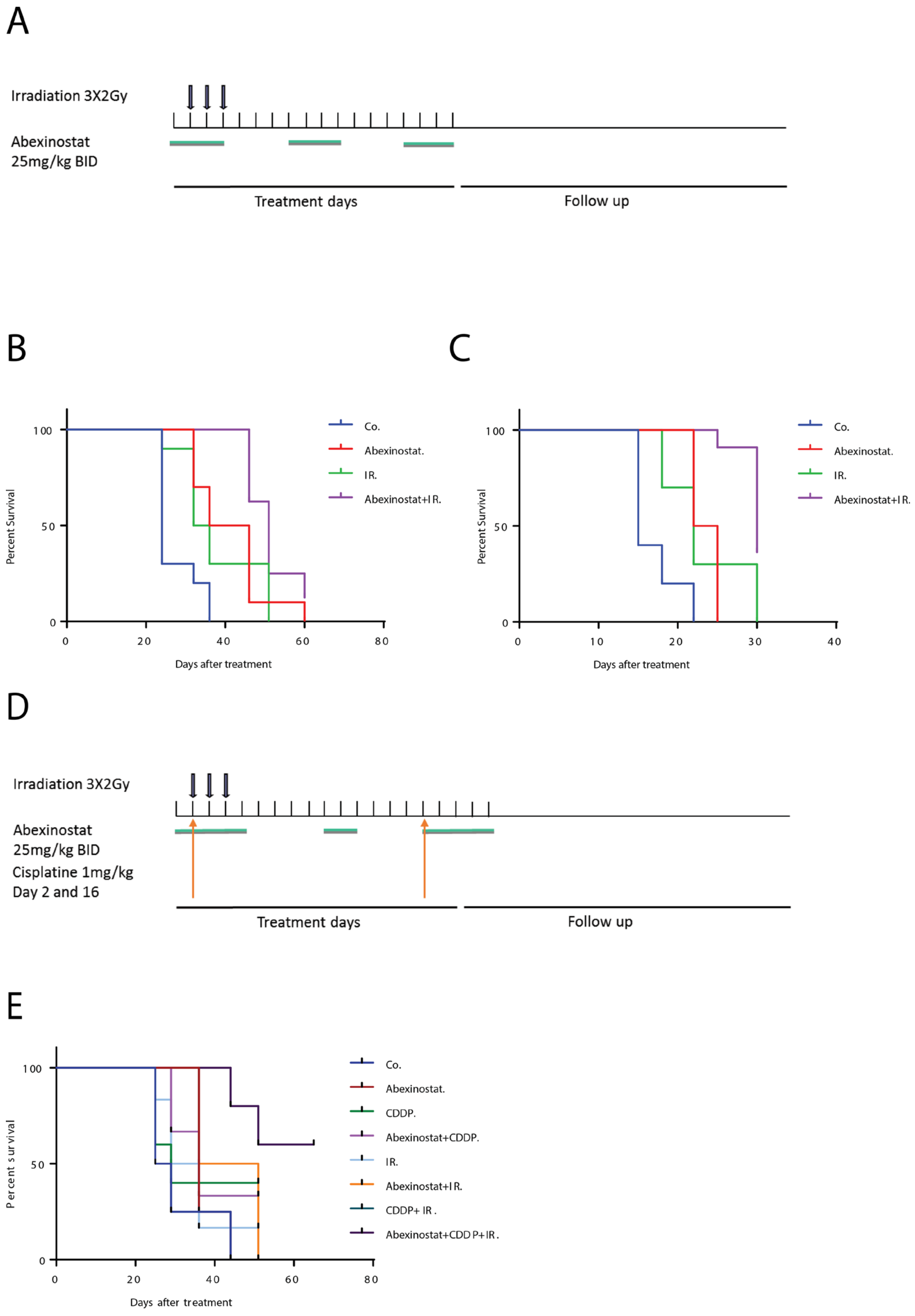

Supplementary Figure 2: *In vivo* treatment schedules and corresponding Kaplan Meier survival curves. Mice bearing A549 or H460 xenografts were treated with +/- abexinostat IP 25mg/kg X 2/d (4 days on, 3 days off, 4 days on, 3 days off, 4 days on) and +/- irradiation at 2Gy/d on day 2, 3 and 4 **A.** Vehicle (HβCD 30%) was used as control. Corresponding Kaplan Meier survival curves for A549 and H460 are represented in **B** and **C.** respectively. Mice bearing A549 xenografted tumors were treated with +/- abexinostat IP 25mg/kg X 2/d (5 days on, 4 days off, 3 days on, 3 days off, 5 days on) +/- Cisplatin IP 1mg/kg 1h before time of irradiation on day 2 and day 16 and +/- irradiation at 2Gy/d on day 2, 3 and 4 **D.** Vehicle (HβCD 30%) was used as control. Corresponding Kaplan Meier survival curve for A549 is represented **E.** Mice were sacrificed after a statistically significant volume difference was reached between groups on day 51 and 65. Therefore a number of data were sensored and we couldn't observe a statistically significant difference in overall survival. A Logrank test for trend was performed and showed a p= 0.0096.

Supplementary Table 1: Abexinostat enhances A549 and H460 cells radiosensitivity in normoxia and hypoxia

	A549 normoxia (21% O_2)				A549 hypoxia (0.1% O_2)			
Conditions	α (Gy^{-1})	β (Gy^{-2})	Mean D_{37} (Gy)	Mean D_{10} (Gy)	α (Gy^{-1})	β (Gy^{-2})	Mean D_{37} (Gy)	Mean D_{10} (Gy)
Control	0.082 ± 0.016 (n=4)	0.022 ± 0.004 (n=4)	5.107 ± 0.33 (n=4)	8.507 ± 0.93 (n=4)	0.113 ± 0.012 (n=2)	-	9.187 ± 2.665 (n=2)	21.15 ± 6.14 (n=2)
Abexinostat 0.4 µM	0.179 ± 0.04 (n=3)	0.022 ± 0.01 (n=3)	3.621 ± 0.29 (n=3)	6.767 ± 0.69 (n=3)	0.2604 ± 0.00 (n=1)	-	3.84 ± 0.00 (n=1)	8.843 ± 0.00 (n=1)
Abexinostat 0.7 µM	0.292 ± 0.04 (n=3)	0.013 ± 0.01 (n=3)	3.078 ± 0.51 (n=3)	6.039 ± 0.62 (n=3)	0.2554 ± 0.025 (n=2)	-	3.938 ± 0.52 (n=2)	9.07 ± 1.19 (n=2)

	H460 normoxia (21% O_2)				H460 hypoxia (0.1% O_2)			
Conditions	α (Gy^{-1})	β (Gy^{-2})	Mean D_{37} (Gy)	Mean D_{10} (Gy)	α (Gy^{-1})	β (Gy^{-2})	Mean D_{37} (Gy)	Mean D_{10} (Gy)
Control	0.199 ± 0.024 (n=4)	0.031 ± 0.007 (n=4)	3.287 ± 0.15 (n=4)	5.976 ± 0.42 (n=4)	0.164 ± 0.014 (n=3)	-	6.416 ± 2.12 (n=3)	14.77 ± 4.87 (n=3)
Abexinostat 0.1 µM	0.359 ± 0.058 (n=2)	0.023 ± 0.018 (n=2)	2.391 ± 0.538 (n=2)	4.812 ± 0.326 (n=2)	0.263 ± 0.02 (n=2)	-	3.78 ± 0.51 (n=2)	8.7 ± 1.17 (n=2)
Abexinostat 0.2 µM	0.312 ± 0.066 (n=3)	0.129 ± 0.03 (n=3)	1.977 ± 0.26 (n=3)	3.227 ± 0.25 (n=3)	0.485 ± 0.05 (n=3)	-	2.03 ± 0.86 (n=3)	4.675 ± 1.98 (n=3)

Alpha and beta values for A549 and H460 cell lines in normoxia and hypoxia and corresponding mean irradiation doses in gray (Gy) to achieve 37% and 10% survival (respectively mean D37 and mean D10) were calculated based on clonogenic survival assays performed as described under materials and methods. For each experiment, the mean D10 dose was calculated using the mean values of alpha and beta determined from the curves drawn for best fit to the experimental data. In each case, the correlation coefficient was more than 0.99 Results represent means ± SD (n= 2 to 5 as indicated in the table).
